# Construction and validation of nursing diagnoses for people with diabetic foot ulcers*

**DOI:** 10.1590/1980-220X-REEUSP-2022-0022en

**Published:** 2022-05-04

**Authors:** Halene Cristina Dias de Armada e Silva, Sonia Acioli, Patricia dos Santos Claro Fuly, Maria Miriam Lima da Nóbrega, Silvia Maria de Sá Basílio Lins, Harlon França de Menezes

**Affiliations:** 1 Universidade do Estado do Rio de Janeiro, Faculdade de Enfermagem, Programa de Pós-Graduação, Rio de Janeiro, RJ, Brazil.; 2 Universidade do Estado do Rio de Janeiro, Faculdade de Enfermagem, Departamento de Enfermagem em Saúde Pública, Rio de Janeiro, RJ, Brazil.; 3 Universidade Federal Fluminense, Escola de Enfermagem Aurora de Afonso Costa, Departamento de Enfermagem Médico Cirúrgico, Niterói, RJ, Brazil.; 4 Universidade Federal da Paraíba, Departamento de Enfermagem de Saúde Pública e Psiquiatria, João Pessoa, PB, Brazil.; 5 Universidade do Estado do Rio de Janeiro, Faculdade de Enfermagem, Departamento de Fundamentos de Enfermagem, Rio de Janeiro, RJ, Brazil.; 6 Hospital Pró-Cardíaco, Rio de Janeiro, RJ, Brazil.

**Keywords:** Nursing Process, Standardized Nursing Terminology, Nursing, Diabetes Mellitus, Diabetic Foot, Primary Health Care, Proceso de Enfermería, Terminología Normalizada de Enfermería, Enfermería, Diabetes Mellitus, Pie Diabético, Atención Primaria de Salud, Processo de Enfermagem, Terminologia Padronizada em Enfermagem, Enfermagem, Diabetes Mellitus, Pé Diabético, Atenção Primária à, Saúde

## Abstract

**Objective:**

To construct and validate nursing diagnoses statements of the International Classification for Nursing Practice (ICNP^®^) for the person with diabetic foot ulcer being followed up in primary health care.

**Method:**

This is a methodological study structured in four stages: identification of terms; cross-mapping of identified terms with ICNP terms^®^, version 2019/2020; construction of nursing diagnoses statements and organization with Orem’s Theory of Self-care; and content validation by expert nurses working in primary care, with those with Content Validity Index (CVI) ≥ 0.80 being considered valid.

**Results:**

Eighty-one diagnostic statements were constructed, five of which were positive, 67 negative, and nine risky. Of these, 58 were included in ICNP^®^ and 23 were not, 51% of which were categorized as self-care requirements related to health changes.

**Conclusion:**

ICNP^®^ subsidized the construction of a technical product, which can be consulted and used by nurses and will allow the strengthening of the standardization of a specific language in the context of care for people with diabetic foot ulcers in primary health care.

## RESUMO

**Objetivo:**

Construir e validar enunciados de diagnósticos de enfermagem da Classificação Internacional para a Prática de Enfermagem (CIPE^®^) para a pessoa com úlcera de pé diabético em acompanhamento na atenção primária à saúde.

**Método:**

Estudo metodológico, estruturado em quatro etapas: identificação de termos; mapeamento cruzado dos termos identificados com os termos da CIPE^®^, versão 2019/2020; construção dos enunciados de diagnósticos de enfermagem e organização com a Teoria do Autocuidado de Orem; e validação de conteúdo por enfermeiros peritos atuantes na atenção primária, sendo considerados válidos aqueles com Índice de Validade de Conteúdo (IVC) ≥ 0.80.

**Resultados:**

Foram construídos 81 enunciados de diagnósticos, sendo cinco positivos, 67 negativos e nove de risco. Desses, 58 eram constantes na CIPE^®^ e 23 não constantes, sendo 51% categorizados como requisitos de autocuidado relativos às alterações de saúde.

**Conclusão:**

A CIPE^®^ subsidiou a construção de um produto técnico, passível de consulta e utilização pelos enfermeiros, o qual permitirá o fortalecimento da padronização de uma linguagem própria no contexto do cuidado à pessoa com úlcera do pé diabético na atenção primária à saúde.

## RESUMEN

**Objetivo:**

Construir y validar enunciados de diagnósticos de enfermería de la Clasificación Internacional para la Práctica de Enfermería (CIPE^®^) para el paciente con úlcera del pie diabético en acompañamiento en la atención primaria a la salud.

**Método:**

Estudio metodológico, estructurado en cuatro etapas: identificación de términos; mapeo cruzado de los términos identificados con los términos de la CIPE^®^, versión 2019/2020; construcción de los enunciados de diagnósticos de enfermería y organización con la Teoría del Autocuidado de Orem; y validez de contenido por enfermeros expertos actuantes en la atención primaria, siendo considerado validos aquellos con Índice de Validez de Contenido (IVC) ≥ 0.80.

**Resultados:**

Fueron construidos 81 enunciados de diagnósticos, siendo cinco positivos, 67 negativos y nueve de riesgo. De esos, 58 eran constantes en la CIPE^®^ y 23 no constantes, siendo un 51% categorizados como requisitos de autocuidado relativos a las alteraciones de salud.

**Conclusión:**

La CIPE^®^ subvencionó la construcción de un producto técnico, que se puede consultar y utilizar entre los profesionales de enfermería, el cual permitirá el fortalecimiento de la estandarización de un lenguaje propio en el contexto del cuidado al paciente con úlcera del pie diabético en la atención primaria a la salud.

## INTRODUCTION

Diabetes mellitus is a heterogeneous metabolic disorder whose main finding is chronic hyperglycemia, and it comprises chronic non-communicable diseases (CNCDs), considered one of the greatest public health problems. The World Health Organization (WHO) reveals that there is growing awareness and concern about the large and growing burden of diabetes, whose global age-adjusted prevalence of cases among adults over 18 has increased from 4.7% in 1980 to 8.5% in 2014^(1)^.

Currently, more than 420 million people live with diabetes worldwide. This number is estimated to increase to 570 million in 2030 and to 700 million in 2045. Brazil is the fifth country in terms of incidence of diabetes in the world, with 16.8 million adult patients (20 to 79 years old). The estimated incidence of the disease in 2030 reaches 21.5 million^(2)^.

These data reveal the magnitude of the clinical condition, leading to the possibility of complications that could be prevented through the diagnosis and adequate management of the disease^(2)^. Among the chronic complications, ulceration and extremities amputation, resulting from the worsening of the diabetic foot, are some of the most serious, with high frequency and socioeconomic impact. The diabetic foot is a syndrome characterized by ulceration, infection, and/or destruction of deep tissues, usually associated with neurological dysfunctions and peripheral vascular disease^(3)^.

In this setting, nurses, who belong to the Primary Health Care (PHC) team, have a key role as they can implement, through the integrality and longitudinality of care, nursing diagnoses (NDs), as they inferred the affected human needs from the data collected from people, and applied them using their judgment as their basis and connecting clinical, social, and behavioral data^(4)^. Therefore, in this context, the use of standardized language systems and the conduction of studies such as this one are relevant, since they allow interdisciplinary communication, facilitate the assessment of the quality of care, promote patient safety, and provide the development of Nursing as a science^(5)^.

Among the main systems, the International Classification for Nursing Practice (ICNP^®^) stands out because it is seen as a standardized terminology that names, classifies, and links phenomena that describe the essential elements of professional practice, namely, nursing diagnoses, outcomes, and interventions, and can be applied to support clinical reasoning, as well as organize the conditions for carrying out adequate nursing care^(6)^.

From this perspective, ICNP^®^ allows grouping terms that lead to the essential elements of the specialized areas. Thus, this study is warranted given that, so far, there have been no productions developed for this priority^(5)^. In addition, the theoretical support adopted in this study, the one by Dorothea Orem’s self-care, is shown to be adequate, as it involves issues related to the need for self-care developed by the person, their family, and community, and the support from the health team in monitoring and preventing the diabetic foot.

The theoretical model by Orem proposes three articulated theoretical bases: Self-Care Deficit Theory, demonstrating the need for intervention when the individual is not competent to perform self-care; Self-Care Theory, whose care practice is performed by the individual with disabilities to maintain his/her life, health and well-being, with the concepts of self-care, self-care demand, therapeutic self-care demand, and self-care requirements being defined; and Theory of Nursing Systems, which is based on the patients’ needs and capabilities to perform self-care, which will determine whether or not to use the intervention of nursing professionals^(7)^.

Based on these concepts, Orem’s model encompasses the complexity involved in caring for people with diabetic foot ulcers, as there is a definition of health deviation requirements that must be met and classifies the Nursing system to be adopted for each individual^(8)^. This way, the nurse works as a regulator of the self-care system, raising current and potential responses, interpreting them clinically, and promoting possible actions.

Therefore, the study aimed at constructing and validating statements of nursing diagnoses of the International Classification for Nursing Practice (ICNP^®^) for the person with diabetic foot ulcer being followed up in primary health care facilities.

## METHOD

### Design of Study

Methodological study, based on content validation, carried out between August 2019 and December 2020 and developed in four stages: 1) Identification of terms; 2) Cross-mapping of terms identified with ICNP terms^®^, version 2019/2020; 3) Construction of nursing diagnoses statements; and 4) Content validation of nursing diagnosis statements by expert nurses working in primary care.

### Study Protocol

In the first stage, for the identification of terms, carried out by the main investigator, a survey of nursing empirical evidence for the care of people with diabetic foot in PHC was conducted, with an integrative literature review and search in official documents in the area being carried out. A review was carried out in the databases *Medical Literature Analysis and Retrieval System Online* (MEDLINE), Latin American and Caribbean Literature on Health Sciences Information (LILACS), as well as in *Cumulative Index to Nursing and Allied Health Literature* (CINAHL), through different combinations of the health descriptors “Diabetic Foot” and “Nursing”, using boolean operators.

The publications selected underwent a process of adaptation, with the removal of sections with low potential for relevant terms, such as titles, authors, acknowledgments, abstracts, methodology, references, footnotes, and information about the authors, as well as official documents. Of these, other complications from diabetes that were not related to diabetic foot were removed.

The articles and documents that were in other languages were fully translated into Portuguese, by a proficient translator, for later unification to the articles and documents in Portuguese, and the publications were grouped in a single Word^®^ file with subsequent conversion to portable document format PDF, which became the corpus of the study at this moment of the research.

The terms were extracted with the support of a computational tool called PorOnto^(9)^, which processed a list in Excel^®^ of terms according to the frequency of appearance. The terms were arranged in alphabetical order for better visualization, with subsequent normalization and standardization of inflections of gender, number, and degree of comparison, to identify and remove term repetitions.

With these terms in hand, the second stage was carried out, when the normalized terms were subjected to cross-mapping with ICNP^®^ and its current version, 2019/2020. Terms/concepts found in the literature were manually mapped with the primitive terms/concepts of the ICNP^®^ Seven Axis Model, with focus on their definitions, to compare them and establish semantic equivalence and exclusion of synonyms. In cases of semantic doubts, a Portuguese dictionary was used^(10)^, to compare to the definitions of ICNP^®^, to reduce difficulties and/or avoid misinterpretation.

The use of the *International Organization for Standardization* (ISO) 12300:2016 was required, as it addresses the standards for mapping terminological systems, providing subsidies for the creation of clinical terminologies or subsets for specific use. The mapping result generated a new Excel spreadsheet^®^ with constant and non-constant primitive concepts in the 2019/2020 version of ICNP^®^.

The terms not included were subjected to an analysis process regarding the similarity and scope in relation to the terms contained in the ICNP^®^ 2019/2020, according to the following criteria^(11)^: the ICNP^®^ term is similar to the identified term, when there is no agreement in spelling, but its meaning is identical; the term is more comprehensive, when it has a broader meaning than the term included in the ICNP^®^; the term is more restricted, when it has a narrower meaning than the one included in the ICNP^®^, and there is no agreement when the term is totally different from the term included in the ICNP^®^.

For the third stage, the nursing diagnoses statements were constructed, using the following empirical evidence: list of specialized nursing language terms for people with diabetic foot ulcers in primary care; ICNP^®^ seven-axis model, version 2019/2020; ISO standard 18.104:2014; Orem’s theoretical model; ICNP^®^ nursing diagnoses/results list, version 2019/2020. For the construction of the diagnoses statements, a term from the “Focus” Axis and a term from the “Judgment” Axis, of a single descriptor equivalent to Focus and Judgment, were included; or just a Clinical Finding that could represent an altered state, an altered function, or even a change in behavior.

The constructed statements were inserted into an Excel spreadsheet^®^, being normalized based on the ICNP^®^ version 2019/2020. Then, they were submitted to the manual cross-mapping process, resulting in a list with NDs included and not included in the ICNP^®^ version 2019/2020. For non-constant statements, an analysis process was carried out regarding the similarity and scope of the ICNP^®^ pre-combined concepts, using the following criteria^(11)^: if the ICNP^®^ statement is similar to the identified one; whether it is more comprehensive; if it is more restricted; or if there is no agreement, thus being a new diagnosis statement.

The diagnoses statements were allocated according to the requirements of Orem’s Self-care Theory, as follows: diagnoses classified according to universal self-care requirements; developmental self-care requirements; and self-care requirements related to health changes^(7)^.

The fourth stage was the content validation by specialist nurses. The distribution of the questionnaires took place by sending a notebook to the experts, in May 2020, by *email*, with instructions on how to fill it out. This included a Letter of Invitation for Participation in the Study; the Free and Informed Consent Form (FICF) in two copies; the expert characterization tool; and the data collection instrument for content validation with diagnostic statements. The time for returning with the answers was 30 days.

### Selection of Experts

For the stage of content validation of the nursing diagnoses statements, we searched for expert nurses working in 35 primary care units of a programmatic area (AP – division in areas for better management of health services) in the city of Rio de Janeiro, as well as those working in the health coordination of the AP mentioned. Nurses were selected through a search in the National Registry of Health Facilities.

The inclusion criteria were: nurses with a minimum of two years of experience in primary care, working in management or care, who are linked to the diabetes program of their units; and knowledge about language systems/nursing diagnoses. The exclusion criterion was being on a leave.

The initial sample included 41 nurses invited to participate in the study, with the final sample being represented by 21 expert nurses, characterizing 51.2% of the invited workers. Nurses who did not fill out the entire instrument or who did not respond the email in the established period were considered dropouts.

### Data Analysis and Treatment

For data analysis, the Content Validity Index (CVI) was used. Therefore, indices were calculated for the scores attributed by the experts to each ND, based on a five-point Likert scale (1 = not pertinent; 2 = slightly pertinent; 3 = very pertinent; 4 = pertinent; 5 = extremely pertinent), with the nursing diagnoses with CVI ≥ 0.80 being validated.

### Ethical Aspects

For the study feasibility, authorization was requested from the Research Ethics Committee of the Universidade do Estado do Rio de Janeiro, with approval through Opinion number 3.501.447/2019, following the standards of Resolution 466/2012, of the National Health Council.

## RESULTS

The first stage of the study resulted in a sample of 62 articles. Five official documents were also used, with two from the Brazilian Ministry of Health, one from the Brazilian Society of Diabetes, one from the Portuguese Ministry of Health and one from the Peruvian Ministry of Health. These documents were chosen because they are reference guides for multidisciplinary health teams in the care of people with diabetes and/or diabetic foot, in the different care settings of the care network, being the most recent publications from these countries.

The extraction of terms found in the productions for the diabetic foot ulcer patient resulted in 12,696 terms, which underwent exclusion of repetitions, normalization and standardization in relation to the ICNP^®^. At the end of these procedures, 392 related terms remained, with 305 nouns, 39 adjectives, and 48 verbs.

Based on these data, a list was built with 98 statements of nursing diagnoses/outcomes (NDs/NOs) for people with diabetic foot ulcers in primary care, which underwent similarity analysis, with repetitions being removed according to their definition. Thus, 81 statements were maintained, five (6%) being positive, nine (11%) risky, and 67 (83%) negative. Of the 81 statements of nursing NDs/NOs built, 58 (71%) are included in the ICNP^®^ as combined diagnoses, present in the Focus axis, or similar to the statements included, and 23 (29%) are not included in the classification.

Among the 23 statements not included in the ICNP^®^, 17 (21%) were classified as more restricted and six (7%) as having no agreement regarding the NDs/NOs or terms from the Focus axis of the classification. There were no statements classified as more comprehensive. All diagnoses were validated by the experts, considering CVI ≥ 0.8 in the general average.

The analysis of nurses’ data showed that 56 (69%) of the diagnostic statements had CVI = 0.9; with 22 (27%) classified with CVI = 0.8 and three (4%) classified with CVI = 1. The statements of NDs/NOs with CVI = 1 were “Hypoglycemia”, “Overweight” and “Nail Care Regimen, impaired”, demonstrating the importance of these diagnoses in the practice of PHC workers.

A relevant fact evidenced was that, among the six statements showing no agreement with the ICNP^®^ statements, five were classified by nurses with CVI = 0.9, namely: “Blister”, “Callus”, “Hair Growth, Absent”, “Maceration” and “Interdigital Moisture, Increased”, and one with CVI = 0.8, being “Skin color, Altered”. This demonstrates that such statements are part of the daily practice of professionals and that they should be included in the classification in the future, to contribute to its improvement.

The diagnoses were classified according to Orem’s self-care requirements, presented in Chart 1, where: 7% of the diagnoses constructed correspond to the universal requirements of self-care; 51%, self-care requirements related to health alterations; and 42%, self-care requirements related to development.

**Chart 1:** Distribution of nursing diagnoses in people with diabetic foot ulcers in primary care according to self-care requirements – Rio de Janeiro, RJ, Brazil, 2022.

Nursing diagnoses	
**Universal self-care requirements**	Feeding, Impaired
	Family Support, Positive
	Ability to Socialize, Impaired
	Cognition, Impaired
	Social Support, Effective
	Sexual Performance, Impaired
**Self-care requirements related to health changes**	Blister
	Callus
	Wound Healing, Impaired
	Skin Color, Changed
	Pain Control, Effective
	Hair growth, Absent
	Pain
	Peripheral Edema
	Balance, impaired
	Erythema
	Fatigue
	Weakness
	Foot Pulse Frequency, Low
	Peripheral Neurovascular Function, Impaired
	Bruise
	Hyperglycemia
	Hyperthermia
	Hypoglycemia
	Infection
	Inflammation
	Skin Integrity, Impaired
	Maceration
	Gait, impaired
	Metabolism, Impaired
	Skin, Dry
	Peripheral Tissue Perfusion, Impaired
	Blood Pressure, Changed
	Plantar Pressure, Severe
	Musculoskeletal System Process, Impaired
	Chill Risk
	Risk of Pedal Pulse Frequency, Absent
	Risk of Hyperthermia
	Risk of Fall
	Risk of Tachycardia
	Bleeding Ulcer
	Stress Overload
	Overweight
	Sleep, impaired
	Diet Tolerance, Impaired
	Diabetic ulcer
	Sight, Impaired
**Developmental self-care requirements**	Acceptance of Health Condition, Impaired
	Adaptation, Impaired
	Alcoholism
	Anxiety
	Attitude towards Care, Positive
	Self-image, Negative
	Low self-control
	Community Ability to Manage the Regimen, Impaired
	Ability to Perform Hygiene, Impaired
	Ability to Perform Leisure Activity, Impaired
	Ability to Participate in Care Planning, Impaired
	Aggressive Behavior
	Communication, impaired
	Risky Housing Condition
	Health Knowledge, Impaired
	Knowledge about Diagnostic Test, Impaired
	Conflicting Cultural Belief
	Religious Belief, Conflicting
	Self-Care Deficit
	Expectation about Treatment, Unrealistic
	Fear
	Non-Adherence to the Physical Exercise Regimen
	Non-Adherence to the Therapeutic Regimen
	Need for Care, High
	Emotional Problem
	Nail Care Regimen, Impaired
	Income, Inadequate
	Responsiveness to Treatment, Low
	Risk of Self-Destructive Behavior
	Quality of Life Risk, Negative
	Suicide Risk
	Suffering
	Smoking
	Interdigital Humidity, Increased

## DISCUSSION

In PHC, the most essential type of care provided to the patient, where the first care with interventions is given, there must be health professionals who provide care aimed at patient’s self-care, and they shall be trained to promote a correct assessment and, subsequently, provide the best treatment and guidelines for the proper care of diabetic foot ulcers, aiming at reducing DM-related morbidity and its complications, as well as ensuring knowledge through patient education and awareness raising^(12)^.

Therefore, the treatment of “Calluses” and “Blisters” as early as possible is recommended, as they are pre-ulcerative lesions that can lead to the appearance of ulcers and/or their increase. Patients’ instruction by professionals regarding the proper drying of the feet is also recommended, to avoid “Interdigital Moisture”, as well as the orientation not to use plasters and substances that generate “Maceration”, since they increase the risk of developing ulcers^(13)^.

Nurses have to know and monitor risk factors leading to diabetic foot complications. Among the main risk factors is peripheral vascular disease, which causes “Absence of Hair Growth” and “Skin Color Change”. When checking for vascular alterations, on inspection, the skin may be atrophic and shiny, with reduced or absent hair, cold extremities and thick nails, and the lower limbs may be pale on elevation and reddened when down^(14)^.

Among the positive diagnoses in the category of universal self-care requirements were “Family Support, Positive” and “Social Support, Effective”. Support from family, friends and the community is essential for the treatment of people with diabetic foot ulcers, as they, due to lack of knowledge or psychological changes, may present inappropriate behavior, maximizing the complications of the disease, and this network is essential in the prognosis of this pathology^(15)^.

Concerning the self-care requirements related to health changes, the nursing consultation, which is exclusive to the nurse, shall be highly accurate to identify risks of ulcerations through rigorous inspection and palpation of dermatological, musculoskeletal, vascular, and neurological changes. To detect dermatological changes, skin thickening (keratosis), calluses, fissures, dry skin, blisters, active ulcers, nail changes, maceration, and interdigital fissures shall be investigated^(16)^.

In the long term and in conditions of poor metabolic control, diabetic foot ulcers can lead to amputation of the extremities, significantly reducing patients’ quality of life, generating physical, psychological, and social repercussions, reaching more than one million cases per year^(17)^. People with hypertriglyceridemia, infection, peripheral arterial disease, and HbA1c (glycated hemoglobin) ≥8 mmol/mol are recognized as being at high risk for developing diabetic foot ulcers as well as for extremity amputations^(18)^, in addition to factors such as trauma, neuropathy and deformity^(19)^.

Another extremely relevant issue is the attention to the patient’s possible visual difficulty due to diabetic retinopathy, as this, associated with cognitive deficit and/or restriction of movements due to physical conditions, such as obesity, hinders the investigation of the own foot, so the person should be helped by other people, such as family members^(20)^.

Some nursing diagnoses presented in the category of self-care requirements regarding development are related to emotional issues from the disease, and nurses should be aware of this dimension of care, since glycemic control is influenced by psychological issues^(21)^, requiring, in addition to control with hypoglycemic agents and changes in lifestyle, the use, in severe cases, of antidepressants.

Other nursing diagnoses are related to the patient’s deficient knowledge about the disease itself and its risks, non-adherence to treatment, low self-control, impaired ability to participate in care planning. In this regard, nurses should maximize their health education actions aiming at promoting health and preventing complications. A study carried out in Cuba with amputee patients due to diabetes complications showed that 70.8% of patients did not have a periodical outpatient follow-up, and less than 32% received health education guidelines for diabetes, not knowing how to accurately and concretely express the necessary foot care, developing harmful actions in self-care^(22)^.

The analysis of patients’ knowledge and adherence to preventive care for the diabetic foot showed that 50% had an impaired degree of mobility; 85% wore inappropriate shoes; and 62.5% removed cuticles, with the presence of mycoses and cracks and high pressure points with lower sensitivity. Data show a divergence between the ideal and real situations, since nurses had not been effective in the self-care education process, as they stated they had provided guidance on the use of suitable shoes and nail clipping^(23)^.

The term “shoe”, although not included in the ICNP’s focus axis^®^, shall be highlighted in this study, as it is considered by several authors as a means of preventing injuries^(2–24)^; however, it is worth mentioning that, due to the socioeconomic condition of the person at risk for diabetic foot ulcers, they may not be able to afford the “best shoe”, being susceptible to injuries or their worsening.

Thus, the term “shoe” shall be considered as fundamental in the care of patients with diabetic foot ulcers, and should be included in other studies for its possible validation as a term of the focus axis, as well as a nursing diagnosis.

A relevant aspect to be mentioned was the fact that we only had two socioeconomic diagnoses statements, namely: “Risky Housing Condition” and “Income, Inadequate”, and 41 NDs/NOs related to self-care requirements regarding health changes, reinforcing the preponderance of care models based on biological indicators that materialize in practical daily care and in research performed by health professionals, including nurses.

To achieve adequate glycemic control among individuals with diabetic foot ulcers, effective primary care, adequate self-care, as well as clear care management strategies involving not only people with ulcers, but also their families and their community and social network, are required. In addition, nurses continuous qualification is important, since many studies show professional disqualification due to lack of training for adequate management of diabetic foot complications.

The representativeness of 29% of diagnoses classified as not included in the ICNP^®^, with six with no agreement with the terms of the ICNP^®^, demonstrates the need for continuity of studies to update this classification, aiming at qualifying assistance in primary care.

Through the use of the theoretical model adopted, it was noticed that the statements of proposed nursing diagnoses addressed the range of care demands, and this allowed, through the classification of statements by self-care requirements, the practical application of this theory in the context of care for the study clientele, which can enhance the relevance of nursing systematization for nursing professionals, ensuring greater visibility and appreciation of the Nursing science.

The specialized nursing terminology constructed in this study, even if not being used daily in clinical practice by nurses in primary care, as evidenced, is shown in the literature, representing routine situations of these professionals, that is, this terminology consists of terms known by nurses.

## CONCLUSION

In this study, 81 statements of diagnoses were constructed, six of which concern universal self-care requirements, 41 are classified as self-care requirements related to health changes, and 34 classified as self-care requirements related to development. The contribution consists of a possible improvement of ICNP^®^, since nurses in primary care shall make the real health needs of the population in their territory evident, using clinical reasoning to build effective nursing diagnoses, essential to support the construction of nursing interventions that ensure access, coordination, comprehensiveness, and longitudinality of care.

## ASSOCIATE EDITOR

Marcia Regina Cubas
